# Virtuelle Realität in der Lehre mit psychisch kranken Patientenavataren

**DOI:** 10.1007/s00115-024-01610-y

**Published:** 2024-01-26

**Authors:** Paraskevi Mavrogiorgou, Pierre Böhme, Marco Kramer, Simon Vanscheidt, Thomas Schoppa, Vitalij Hooge, Nico Lüdike, Thies Pfeiffer, Georg Juckel

**Affiliations:** 1 Klinik für Psychiatrie, Psychotherapie und Präventivmedizin, LWL-Universitätsklinikum, Ruhr Universität Bochum, Alexandrinenstr. 1–3, 44791 Bochum, Deutschland; 2 Raumtänzer GmbH, Nickelstr. 21, 33378 Rheda-Wiedenbrück, Deutschland

**Keywords:** VR, Ausbildung, Studium, Medizin, Psychiatrie, VR, Education, Studying, Medicine, Psychiatry

## Abstract

**Hintergrund:**

Ärztliche Interaktions- und Explorationstechniken sind die wichtigsten Werkzeuge, die Medizinstudierende im Fach Psychiatrie und Psychotherapie zu erwerben haben. Die aktuell verfügbaren modernen digitalen Technologien wie Virtual Reality (VR) können als wichtige Ergänzungen zu einer Verbesserung der Vermittlung psychiatrisch-psychopathologischer Lerninhalte sowie Diagnosestellung beitragen.

**Ziel der Arbeit:**

Evaluation des Bochumer Avatar-Explorationsprojektes (AVEX) im Rahmen des kurrikularen Kurses im Medizinstudium an der Ruhr-Universität Bochum auf dessen Möglichkeiten zur Vermittlung von Lerninhalten und Techniken der psychiatrischen Anamnese- und Befunderhebung.

**Material und Methoden:**

Im AVEX konnten bislang insgesamt 87 Medizinstudierende des klinischen Studienabschnitts in den Dialog mit „psychisch kranken“ Avataren, also virtuellen Patienten und Patientinnen, treten und ihre Erfahrungen mit der VR-Technologie als Lern- und Lehrmethode im Fach Psychiatrie und Psychotherapie machen und mittels eines Fragebogens rückmelden.

**Ergebnisse:**

Eine wesentliche Vermittlung von Lerninhalten hinsichtlich des psychopathologischen Befundes ist trotz eingeschränkter Interaktionsmöglichkeiten mit den digitalen Avataren erzielbar. Allerdings müssen die Studierenden hierbei gut durch die Dozierenden unterstützt und auch die technischen Möglichkeiten der Spracherkennung weiter verbessert werden.

**Diskussion:**

Das Projekt AVEX zeigt bereits hoffnungsvolle Möglichkeiten zur Ergänzung der Lehre von Medizinstudierenden auf, auch wenn die Passung von Fragen und Antworten im Dialog mit den virtuellen Avataren noch verbessert werden muss. Da von Fortschritten bei der sprachlichen Vermittlung von Emotionen und den visuellen Effekten der Avatardarstellung auszugehen ist, wird der Stellenwert dieser Technik weiter zunehmen.

## Abstract (en)

**Background:**

Medical interaction and exploration techniques are the most important tools that medical students have to acquire in the subject of psychiatry and psychotherapy. The new digital technologies currently available, such as virtual reality (VR), as important supplements can contribute to a significant improvement in the teaching of psychiatric-psychopathological learning content as well as, in particular, the technique of ascertaining the psychiatric history and diagnosis.

**Objective:**

Evaluation of the Bochum Avatar Exploration Project (AVEX) as part of the curricular course in medical studies at the Ruhr University Bochum for its possibilities to convey learning content and techniques of anamnesis and diagnosis in the subject of psychiatry and psychotherapy.

**Methods:**

In AVEX, a total of 87 medical students in the clinical study section have so far been able to enter into a dialogue with “mentally ill” avatars and gain experience with VR technology as a learning and teaching method in the subject of psychiatry and psychotherapy.

**Results:**

Despite the limited possibilities for interaction with the digital avatars, it is possible to achieve a substantial transfer of learning content in psychiatry; however, the students must be well supported by the lecturers.

**Conclusion:**

The AVEX project already shows promising possibilities for supplementing the teaching of medical students, even if the fit of questions and replies in dialogue with the virtual avatars still needs to be improved. As advances in the linguistic communication of emotions and the visual effects of the avatar representation can be predicted, the significance of this technology will continue to increase.

Bereits lange vor der COVID-19-Pandemie und den damit verbundenen Einschränkungen des Präsenzunterrichts wurde die stärkere Verankerung digitaler Lehr- und Lernangebote beispielsweise mittels virtueller Realität (VR) im Kurrikulum des Medizinstudiums empfohlen [12]. Nach ersten vielversprechenden Ergebnissen aus der medizinischen Ausbildung und klinischen Versorgung in somatischen Fachgebieten wird im vorliegenden Artikel der Einsatz des Bochumer Avatar-Explorationsprojekts (AVEX) in der psychiatrisch-psychotherapeutischen Lehre evaluiert.

## Hintergrund

Der Einsatz von Simulationen in der virtuellen Realität (VR) als Lern- und Lehrmethode im Medizinstudium wächst stetig [3, 6, 19]. Trotz der noch limitierten Hard- und Software bietet VR immer immersivere und interaktivere Lernumgebungen an, die für verschiedene medizinische Fachrichtungen ausdifferenziert werden können [14]. In der vorklinischen Anatomie können 3‑D-Körpermodelle und Präparationskurse in VR gerade angesichts z. B. fehlender Körperspendern effektive Alternativen sein [7, 16]. Trotz berichteter unerwünschter Wirkungen, am häufigsten Kopfschmerzen oder Schwindel, welche aber durch Nutzereingewöhnung nachlassen, ermöglichen VR-Anwendungen gegenüber z. B. tabletbasierten Anwendungen Vorteile wie z. B. ein tieferes „Eintauchen“ in den Lernstoff [16].

In der chirurgischen Lehre sind VR-Anwendungen kaum mehr wegzudenken [2, 8, 13]. Die Durchführung grundlegender chirurgischer Eingriffe durch Medizinstudierende in einem virtuellen Operationssaal ermöglicht das Erlernen wesentlicher operationsrelevanter Kenntnisse und Fähigkeiten ohne Ängste und ohne Ablenkung durch eine potenziell einschüchternde OP-Atmosphäre [13, 26]. Auch zur Vermittlung neuroanatomischer Kenntnisse sowie neurochirurgischer Techniken zeigen sich interaktive VR-Anwendungen effektiv und werden als wertvolle und nützliche Lernerfahrung empfunden [1].

Angesichts dieser vielversprechenden Ergebnisse wurde der Einsatz von VR auch für das Fach Psychiatrie und Psychotherapie diskutiert [4, 20, 24], hat sich aus verschiedenen Gründen jedoch bislang nicht in der Routine von Lehre, Diagnostik und Therapie etabliert [22]. Als hinderlich wirken zum einen die relativ hohen Anschaffungskosten (für einen VR-Arbeitsplatz ein mindestens vierstelliger Betrag), zum anderen die mangelnde Verfügbarkeit und Kosten für in VR geschultes und erfahrenes Unterrichtspersonal. Zudem sind Softwareprogramme zur Vermittlung psychiatrisch-psychotherapeutischer Lerninhalte kaum verfügbar, denn anders als z. B. die virtuelle Abbildung des Herzens ist die Seele weiterhin eine schwer zu fassende „Entität“.

**Figure 1:**
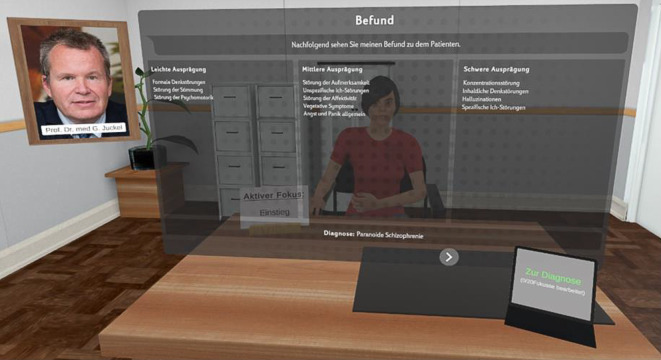

Die in Gesundheitsberufen essenziellen nichttechnischen Fähigkeiten, zu denen neben dem affektiv-emotionalen Austausch auch z. B. Kommunikation und Situationsbewusstsein zählen, lassen in VR-Simulationen durch die technischen Gegebenheiten (noch) nicht wirklich gut umsetzen [3]. Die Entwicklung eines für die Psychiatrie spezifischen VR-Programms mit Verwendung von Avataren, welche psychisch kranke Patienten digital darstellen, zum Erlernen und Üben der strukturierten Erhebung einer psychiatrischen Anamnese und des psychopathologischen Befundes (Bochumer AVEX-Projekt) wurde bereits vorgestellt [15]. Die aktuelle Version unseres VR-Programms ermöglicht es den Studierenden, in einer immersiven virtuellen Explorationssituation verschiedene Avatarpatienten mit unterschiedlichen psychiatrischen Erkrankungen anamnestisch bezüglich biografischer und krankheitsbezogener Aspekte zu befragen und einen psychopathologischen Befund zu erheben, sodass eine diagnostische Einschätzung erfolgen kann. Für die Exploration kam ein Fokusmodus zum Einsatz, der die psychopathologischen Items mit ihren passenden Fragen und Antworten entsprechend dem standardisierten Befundsystem der Arbeitsgemeinschaft für Methodik und Dokumentation in der Psychiatrie (AMDP) in Kategorien gruppiert (z. B. „inhaltliche Denkstörungen“) verwendet. Auf die vom jeweiligen Studierenden gestellten Fragen gibt der jeweilige Avatar spezifische Antworten, sofern die Frage zutreffend im jeweiligen Fokus gestellt wurde, die dann zur Erstellung des Befundes und Verdachtsdiagnose seitens des Explorierenden beitragen. War die Frage nicht zutreffend formuliert, antwortet der Avatar mit Unverständnis und sie muss neu und anders gestellt werden. Am Ende der Exploration erfolgt digital der Abgleich mit dem hinterlegten Befund und der Diagnose, sodass der Studierende unmittelbar ein Feedback zu seinem erhobenen psychopathologischen Befund und zur von ihm gestellten psychiatrischen Diagnose erhält (Abb. 1).

Begleitend zum VR-Unterricht kam routinemäßig ein Fragebogen zur Evaluation zum Einsatz, der differenzierte Erkenntnisse über den Einsatz der Technologie der virtuellen Realität in der psychiatrisch-psychopathologischen Ausbildung von Medizinstudierenden und Rückschlüsse auf die Effektivität der Lehre mit dieser Methode ermöglicht.

## Material und Methoden

### Studierendengruppe

**Figure 2:**
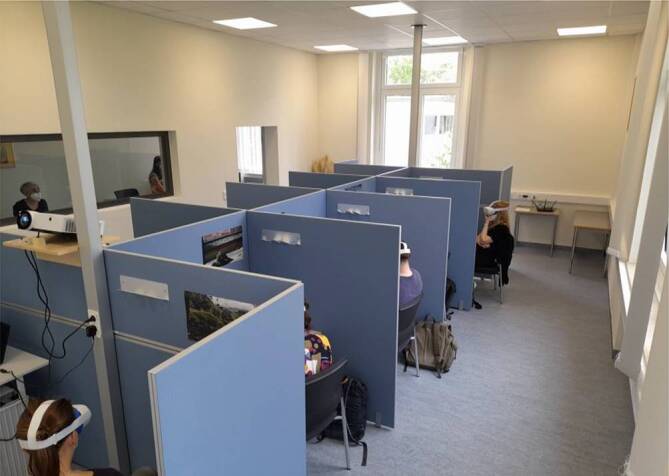

Während des Sommersemesters 2022 (6. Semester) und Wintersemesters 22/23 (7. Semester) nahmen insgesamt 87 Medizinstudierende der Ruhr-Universität Bochum am VR-Unterricht im Rahmen des regulären Psychiatriekurses (UaK) teil. Das VR-Labor der Klinik für Psychiatrie und Psychotherapie des LWL-Universitätsklinikums bietet 10 separate Arbeitsplätze (Kabinen), die jeweils mit der VR-Brille Meta Quest 2 ausgestattet sind (Abb. 2). Nach einer 10-minütigen Einweisung in die Bedienung der VR-Brille sowie des Programms durch Mitarbeitende der Forschungsabteilung für Experimentelle Psychopathologie (EX-PSY) konnten die Studierenden ihre Exploration des Avatars beginnen. Für alle 10 Arbeitsplätze wurde der gleiche psychisch kranke Avatar ausgewählt, der thematisch dem in der aktuellen Hauptvorlesung angesprochenen Krankheitsbild entsprach. Während der 30- bis 40-minütigen Exploration wurden die Studierenden neben ihrem Gruppendozenten auch von Mitarbeitern des VR-Labors betreut und unterstützt. Am Ende erfolgte neben dem Ausfüllen des Evaluationsfragebogens eine je nach Bedarf unterschiedlich lange Nachbesprechung, in der sowohl fallbezogene als auch VR-spezifische Aspekte thematisiert wurden.

### Fragebogen

Der eigens hierfür konzipierte, pseudoanonymisierte Evaluationsfragebogen erfasst als Selbsteinschätzungsinstrument neben Alter und Geschlecht die subjektive Erfahrung mit und Bewertung der VR-Anwendung sowie die Effektivität der Lernzielvermittlung der Medizinstudierenden. Er umfasst 10 Fragen, welche anhand einer visuellen Analogskala bewertet werden (von 1: sehr schlecht bis 5: sehr gut), sowie 3 qualitative Fragen, die fakultativ für persönliche Erlebnisse oder Anmerkungen zum VR-Unterricht genutzt werden konnten („Was hat Ihnen an dem heutigen Termin gut gefallen? Was hat Ihnen nicht gefallen und haben Sie hierfür Verbesserungsvorschläge? Haben Sie sonst noch Anmerkungen?“).

## Auswertung

Die statistische Auswertung der quantitativen Daten erfolgte mittels des Programms IBM SPSS Statistics 27. Zur deskriptiven Analyse erfolgte die Berechnung der Mittelwerte und Standardabweichungen der erhobenen Daten. Die statistische Analyse erfolgte mittels parametrischer und/oder nichtparametrischer Tests (t-Test, Kruskal-Wallis-Test, χ^2^ sowie Spearman-Korrelationskoeffizienten). Werte von *p* ≤ 0,05 wurden als statistisch signifikant, bzw. Werte von *p* ≤ 0,01 als statistisch hoch signifikant gewertet.

Die Analyse der qualitativen Daten erfolgte durch induktive Kategorienbildung und diesbezüglicher deskriptiver Statistiken.

## Ergebnisse

Von den 87 Medizinstudierenden, die am VR-Unterricht teilgenommen hatten, waren 46 % (*n* = 40) männlich und 42,5 % (*n* = 37) weiblich, wobei 10 der Teilnehmenden (11,5 %) keine Angabe zur Geschlechtszugehörigkeit machten. Das mittlere Durchschnittalter lag bei 25,7 Jahren (SD = 4,5 Jahre; Range 20–38), allerdings hatten hier nur 73 von 87 Studierende ihr Alter angegeben. Die männlichen Studierenden waren mit 26,3 Jahren (SD = 4,6) älter als die Frauen mit 25,1 Jahren (SD = 4,3), jedoch war der Altersunterschied nicht statistisch signifikant (*p* = 0,261).

In der Tab. 1 sind die mittleren Werte hinsichtlich der ersten 10 Fragen des Evaluationsbogens dargestellt. Am besten wurde mit einem Durchschnittswert von 4,5 (SD = 0,7) die Einweisung in das VR-Programm bewertet. Am schlechtesten wurde mit einem mittleren Wert von 2,7 (SD = 0,9) die Qualität in Bezug auf die Möglichkeit der eigenen Interaktion mit dem Avatar bewertet. Auffällig war, dass die männlichen Medizinstudierenden die ersten 4 Fragen v. a. in Bezug auf die Vermittlung von Lerninhalten, Präsenz und einer realitätsnahen Darstellung im VR-Raum signifikant kritischer und negativer beantwortet haben als die Medizinstudentinnen (Tab. 1).

**Table 1:** 

Frage: Wie beurteilen Sie …	Gesamt	Männlich	Weiblich	t‑test
1. die Umsetzung des Avatars als Patient, M (SD)	3,4 (0,8)	3,2 (0,9)	3,5 (0,7)	n. s.
2. die Vermittlung von Lerninhalten, M (SD)	3,1 (0,8)	**2,9 (0,8)**	**3,3 (0,8)**	***p*** **=** **0,015**
3. die Qualität der Interaktionsmöglichkeit, M (SD)	2,7 (0,9)	2,5 (0,9)	2,9 (0,8)	*p* = 0,055
4. die Qualität der realitätsnahen Darstellung, M (SD)	3,3 (0,9)	**3,0 (1,0)**	**3,4 (0,8)**	***p*** **=** **0,019**
5. Ihr eigenes Gefühl, in der virtuellen Umgebung selbst präsent zu sein, M (SD)	3,6 (1,0)	3,5 (1,1)	3,6 (1,0)	n. s.
6. die Möglichkeit der VR, Sie beim Lernen im Fach Psychiatrie zu unterstützen, M (SD)	^a^3,6 (1,0)	3,4 (1,2)	3,7 (0,8)	n. s.
7. die Sprachqualität des Avatars, M (SD)	^a^3,9 (0,9)	3,7 (1,0)	4,0 (0,7)	n. s.
8. die Einweisung in die Bedienung im VR-Labor, M (SD)	^a^4,5 (0,7)	4,4 (0,8)	4,6 (0,5)	n. s.
9. die Bedienung, Anwenderfreundlichkeit im virtuellen Raum, M (SD)	^a^3,9 (1,0)	3,9 (1,1)	4,0 (0,8)	n. s.
10. den VR-Unterricht insgesamt, M (SD)	^a^3,4 (0,9)	3,2 (1,0)	3,6 (0,8)	n. s.

*M* Mittelwert, *SD* Standardabweichung

^a^Bei den Fragen 6–10 gab es 2 Teilnehmer, die diese Fragen nicht beantwortet haben

Ein Großteil der Medizinstudierenden (37,9 %) machte keine Angaben zu der Frage, was ihnen am VR-Kurstag gut gefallen hat. Fast 30 % waren besonders von der VR-Methode als solche angetan. Dabei überwogen Aussagen wie „interessante, innovative, abwechslungsreiche neue Lehrmethode“. Positiv wurde von ca. 16 % die Möglichkeit bewertet, im Rahmen des VR-Kurses „ohne Druck eigenständige Exploration“ durchführen zu können. Jeweils 7 % der Studierenden kommentierten die gesamte Organisation des VR-Kurstags positiv sowie die Möglichkeit, im Rahmen des regulären Psychiatriekurses überhaupt VR kennenzulernen und eine neue persönliche Erfahrung machen zu dürfen.

In der Tab. 2 sind die häufigsten Antwortkategorien in Bezug auf die qualitativen Fragen dargestellt. Von einem Drittel der Studierenden wurden keine negativen Aussagen zum VR-Unterricht getroffen. Die häufigsten negativen Aussagen betrafen die technischen Unzulänglichkeiten des Softwareprogrammes wie z. B. „keine tatsächliche flüssige Interaktion“, da nur im Vorhinein implementierte Fragen erkannt wurden und die Antworten darauf teilweise zu lang waren. Auch Spracherkennungsprobleme sowie das Gewöhnen an das Tragen der VR-Brille v. a. in Kombination mit der infektiologisch notwendigen Gesichtsmaske wurden als negative Faktoren genannt. Über 13 % der Studierenden hätten sich eine intensivere Vorbereitung auf den VR-Unterricht (hier insbesondere bezüglich des AMDP-Interviews) und eine längere Explorationsdauer bzw. wiederholende VR-Sitzungen gewünscht.

**Table 2:** 

Frage/Kategorien		Anzahl der Antworten/Aussagen
11**.**	**Gut gefallen**	
	a) Methode	26 (29,9 %)
	b) Selbständigkeit	14 (16,1 %)
	c) Organisation	6 (7,1 %)
	d) Persönliche Erfahrung	6 (7,1 %)
	e) Keine Aussage	33 (37,9 %)
12.	**Nicht gut gefallen**	
	a) Avatar versteht die Fragen nicht	19 (21,8 %)
	b) Antworten des Avatars zu lang, unspezifisch bzw. unterbrechbar	8 (9,2 %)
	c) Organisatorische und formale Rahmenbedingungen	12 (13,8 %)
	d) Eingeschränkte Interaktion	17 (19,5 %)
	e) Keine Aussage	29 (33,3 %)
13.	*Sonstige Anmerkungen*	
	a) Positive	8 (9,2 %)
	b) Negative	14 (16,1 %)
	c) VR kein Ersatz für Unterricht mit echten Patienten	5 (5,7 %)
	d) Keine Aussage	58 (66,7 %)

Unter „Sonstiges“ wurde mehrheitlich (66,7 %) keine Aussagen formuliert. Die positiven Aussagen (9,2 %) unter „Sonstiges“ waren eher unspezifisch wie „alles fein“, „war top“ oder „hat Spaß gemacht“, während unter den rein negativen Anmerkungen (16,1 %) Aspekte formuliert wurden, die die Unzulänglichkeiten des Softwareprogrammes thematisierten, aber eigentlich auf die Unkenntnisse der Studierenden zurückzuführen waren. Beispielsweise kritisierten die Studierenden wiederholt falsche oder fehlende Antworten auf ihre gestellten Fragen, was aber überwiegend auf die Auswahl des falschen Fokus aus mangelnder Kenntnis des AMDP-Befundsystems zurückgeführt werden konnte (z. B. Frage nach „Stimmenhören“ im vom Studierenden gewählten Fokus „Wahn und inhaltliche Denkstörungen“ und nicht im Fokus „Sinnes- und Wahrnehmungsstörungen“, wodurch der Avatar diese nicht richtig beantworten konnte).

Wiederholt wurde von 5,7 % der Studierenden klar formuliert, dass der VR-Unterricht keinen Ersatz für den UaK mit echten Patienten darstellen könne und als solches auch nicht betrachtet werden sollte.

In explorativen Korrelationsanalysen zeigte sich eine inverse Korrelation vom Studierendenalter mit der Qualität der Präsenz im virtuellen Raum (r = −0,250, *p* = 0,033).

Die Qualität der Einweisung in die Bedienung des VR-Systems korrelierte mit der Formulierung positiver Bewertungen (r = 0, 218, *p* = 0,047), während eine subjektiv erlebte schlechte Interaktionsmöglichkeit mit einer negativen Aussage zum VR-Kurstag zusammenhing (r = 0,247, *p* = 0,023). Letztere der hier dargestellten Zusammenhänge bestätigen auch den allgemeinen Eindruck der VR-Labor-Mitarbeitenden, der jedoch nicht standardisiert erfasst wurde. Ebenso wurde das Auftreten von Cybersickness nicht quantitativ erfasst, sondern nur qualitativ notiert. Insgesamt mussten lediglich 2 Studierende (2,3 %; jeweils 1 männlich und 1 weiblich) die VR-Exploration aufgrund eines Übelkeitsgefühls abbrechen und diese an einem Computer fortführen.

## Diskussion

Die ersten Ergebnisse des Bochumer AVEX-Projekts zeigen, dass VR-Unterricht im Rahmen des regulären Psychiatriekurses im Medizinstudium insgesamt mittel bis gut bewertet wird. Vor allem zeigten sich z. T. deutliche Geschlechtseffekte. Jüngere Kursteilnehmerinnen bewerteten die VR-Methode zur Vermittlung psychiatrisch-psychopathologischer Lerninhalte, aber auch bezogen auf die einzelnen technischen Aspekte von VR wie z. B. Immersion, also das Realitätsgefühl, grundsätzlich besser als die männlichen Studierenden.

Über geschlechterspezifische Unterschiede in der Erfahrung und Beurteilung von VR-Anwendungen im Medizinstudium gibt es bislang kaum Berichte. Bezüglich digitaler Spielerfahrungen und Integration neuer Medientechnologien wie beispielsweise Multiplayer-Onlinesimulationen in die medizinische Ausbildung äußerte sich in einer US-Studie von 2010 eine große Mehrheit der Medizinstudierenden positiv [11]. Geschlechtsspezifische Unterschiede bestanden dabei lediglich in Bezug auf die Art der bevorzugten Spiele, den pädagogischen Wert von Videospielen und den Wunsch an Spielen teilzunehmen, wobei dort die Studentinnen insgesamt kritischer in der Bewertung waren [11].

In einer aktuelleren systematischen Übersichtsarbeit mit 22 eingeschlossenen Studien zeigten sich keine signifikanten Geschlechterunterschiede, auch wenn ein allgemeiner Konsens aufgrund der Heterogenität und Unausgewogenheit der Studienpopulationen sowie VR-Anwendungen nicht abzuleiten sei [10]. „Präsenz“ beschreibt das Gefühl des Nutzers, in die virtuelle Welt eingebunden zu sein, und kann somit als das subjektive Erlebnis des Nutzers im Sinne einer emotional-kognitiven psychophysiologische Reaktion und auch als eine „Wahrnehmungsillusion“ verstanden werden, da man die dahintersteckende VR-Technik („Mediatisierung“) nicht mehr wahrnimmt [15, 21]. Unser Ergebnis, dass die Medizinstudentinnen Realitätsnähe, Präsenz, aber auch Interaktionsmöglichkeit besser bewerteten als die männlichen Kursteilnehmer, deckt sich mit anderen Studien [5, 17, 18, 23].

Ein beträchtlicher Teil der hier befragten Medizinstudierenden konnte die VR-Technologie sowohl bezüglich ihrer potenziellen Möglichkeiten als auch ihrer Limitationen realistisch einordnen. Sie betrachteten es als ein innovatives unterhaltsames, abwechslungsreiches, integratives und fesselndes Mittel zum Lernen und waren der Meinung, dass VR die derzeitigen Lehr- und Lernansätze ergänzen, ihr Selbstvertrauen stärken und ihnen einen sicheren Raum (vor den Bewertungen der Dozierenden und der Mitstudierenden) zum Ausprobieren und Problemlösen bieten würde. Andererseits zeigte sich trotz der größeren technischen Versiertheit jüngerer Generationen auch eine Überschätzung der gegenwärtigen technischen Möglichkeiten zur Interaktion mit dem Avatar. Zukünftige Entwicklungen sollten daher stärker menschenähnliche interaktive Interventionen sowie die Entwicklung und Verbesserung emotional-affektiver Avatare umfassen [9, 25].

Im Umgang mit dem AVEX-Programm zeigten Medizinstudierende Schwierigkeiten mit dem eng am psychopathologischen Befund gemäß dem AMDP-System orientierten Fokussystem. Je sicherer die Studierenden im Abfragen der psychopathologischen Dimensionen waren, desto flüssiger und richtiger war die Beantwortung durch den Avatar. Die Vermittlung psychiatrischen Wissens, aber auch der Erfolg des AVEX-Programms ist damit abhängig von einer strukturierten und standardisierten Verwendung AMDP-basierter Merkmale im Sinne einer „gemeinsamen Sprache“.

Auch wenn nicht besonders häufig geäußert, so muss die Aussage unserer Studierenden, dass der VR-Unterricht nicht als Ersatz für den Unterricht mit echten Patienten und Patientinnen betrachtet werden sollte, hier abschließend gewürdigt werden. Eine mögliche Erklärung ist ein durch die COVID-19-Pandemie verringerter Präsenzunterricht der eingeschlossenen Studierenden und die dadurch verstärkte Verunsicherung, nicht gut genug für die Ausübung des Arztberufes vorbereitet zu werden. Nichtsdestotrotz bewerteten die Teilnehmenden unseres VR-Unterrichts die VR-Technologie als innovative Ressource in der Psychiatrielehre, zeigten aber auch die Notwendigkeit einer Unterstützung und eines kontinuierlichen persönlichen Feedbacks auf.

Obgleich die Protokollfunktion zu dem Zeitpunkt der Evaluation noch nicht ausgereift war, haben durchaus vergleichbar mit dem analogen Unterricht ca. 90 % der Studierenden die richtige Diagnose gefunden. Dies und den zutreffenden psychopathologischen Befund erhielten die Studierenden jeweils am Ende des Programms. Zukünftig wird es möglich sein, nach mehrfacher Nutzung des Programms individuelle Lernkurven jedem Studierenden zurückzumelden. Auch müsste der durch das Studienteam konzipierte und noch nicht validierte Evaluationsfragebogen, der bei dieser Evaluation sicherlich ein einschränkender Faktor war, weiter überarbeitet werden.

Insgesamt kann der Einsatz von VR trotz der noch eingeschränkten Interaktion auch im Fach Psychiatrie dabei helfen, ärztliche Fertigkeiten zu üben und medizinisches Wissen zu erlangen. Das hier vorgestellte Projekt AVEX zeigt hoffnungsvolle Möglichkeiten bei der Ergänzung der Lehre und dem Unterrichten von Medizinstudierenden. Angst vor einem Verlust der Daseinsberechtigung als unterrichtender Mediziner, da die Technik ihn ersetzen könnte, oder davor, keine echten Patienten mehr in der Lehre zu erleben, erscheint uns dabei fehl am Platz, sondern die VR wird den Weg zu einem immer effizienteren Lehren und Lernen stärken. Hierfür sind jedoch interdisziplinäre Kooperationen notwendig, um diese zukunftsweisende digitale Technologie zu verbessern.

## Fazit für die Praxis

- Virtuelle psychisch kranke Avatare können die Lehre im Fach Psychiatrie und Psychotherapie bereichern.
- Die Evaluationen durch Studierende sind trotz noch bestehender Limitationen insgesamt positiv.
- Notwendige Weiterentwicklungen umfassen vor allem eine Verbesserung der interaktiven Potenziale der Avatare.
- Interdisziplinäre Kooperationen sind notwendig, um komplexe Projekte mit virtuellen Avataren zu realisieren und zu optimieren.
